# Effectiveness, Tolerability, and Safety of Belimumab in Patients with Refractory SLE: a Review of Observational Clinical-Practice-Based Studies

**DOI:** 10.1007/s12016-018-8675-2

**Published:** 2018-03-06

**Authors:** Francesca Trentin, Mariele Gatto, Margherita Zen, Larosa Maddalena, Linda Nalotto, Francesca Saccon, Elisabetta Zanatta, Luca Iaccarino, Andrea Doria

**Affiliations:** Division of Rheumatology, University of Padova, Via Giustiniani, 2, 35128 Padova, Italy

**Keywords:** Systemic lupus erythematous, Belimumab, BLyS, Drug survival, Drug efficacy, Real-life experience

## Abstract

To date, belimumab is the only biological drug approved for the treatment of patients with active refractory SLE. We compared and critically analyzed the results of 11 observational clinical-practice-based studies, conducted in SLE referral centers. Despite the differences in endpoints and follow-up duration, all studies remarked that belimumab provides additional benefits when used as an add-on to existing treatment, allowing a higher rate of patients to reach remission and to taper or discontinue corticosteroids. In the OBSErve studies, 2–9.6% of patients discontinued corticosteroids and 72–88.4% achieved a ≥ 20% improvement by physician’s judgment at 6 months. In Hui-Yuen’s study, 51% of patients attained response by simplified SRI at month 6. In Sthoeger’s study, 72.3% of patients discontinued corticosteroids and 69.4% achieved clinical remission by PGA after a median follow-up of 2.3 years. In the multicentric Italian study, 77 and 68.7% of patients reached SRI-4 response at months 6 and 12, respectively. In all the studies, disease activity indices decreased over time. Retention rates at 6, 9, and 12 months were 82–94.1, 61.2–83.3, and 56.7–79.2%, respectively. The main limitations of these studies include the lack of a control group, the short period of observation (6–24 months) and the lack of precise restrictions regarding concomitant medication management. This notwithstanding, these experiences provide a more realistic picture of real-life effectiveness of the drug compared with the randomized controlled clinical trials, where stringent inclusion/exclusion criteria and changes in background therapy could limit the inference of data to the routine clinical care.

## Introduction

Systemic lupus erythematosus (SLE) is a chronic, autoimmune disorder of unknown etiology that can virtually involve any organ system of the body.

Conventional drugs for the treatment of SLE include antimalarials, non-steroidal anti-inflammatory drugs (NSAIDs), corticosteroids, and immunosuppressants. Despite the advances in the treatment, patients with SLE still have an unsatisfactory long-term prognosis. Due to persistent disease activity or disease flares, a high percentage of patients require long-term corticosteroid and/or immunosuppressive treatment, which leads to progressive damage accrual and worsening of *quoad vitam* and *quoad valetudinem* prognosis [1].

Recent studies on SLE treatment have mainly been focused on two directions: new therapeutic strategies, such as treat-to target [2, 3], and new targeted therapies.

Unlike rheumatoid arthritis and spondyloarthritis, biologic therapy is still limited in lupus. Several biologics have been tested in recent years, targeting both the adaptive and the innate immune system. Despite the encouraging evidences provided by case series and clinical-practice-based uncontrolled studies, the majority of the randomized controlled trials (RCTs) did not achieve the primary endpoint, thereby committing physician to off-label use of targeted therapies in SLE [4–8]. To date, the only biological drug approved by the Food and Drug Administration (FDA) and the European Medicines Agency (EMA) is belimumab (Benlysta®, GSK, United Kingdom), a fully-human IgG1-λ monoclonal antibody which selectively binds and inhibits soluble BLyS.

BLyS (B lymphocyte stimulator) is a 250 amino acid protein that plays an important role in the development, selection, and survival of B cells [9–13]. The rationale behind indirect targeting of B cells by BLyS, rather than direct targeting by a CD20-based approach, is to preferentially suppress pathogenic B cells, without affecting the protective role of lymphocytes against infections. There are indeed strong evidences, both in mice [14, 15] and in humans [16–19], that BLyS could contribute to the loss of tolerance in SLE: The excess concentration of BLyS inhibits the physiological apoptosis of low-affinity self-reactive B cells and promotes their pathological differentiation into autoantibody-producing plasma cells [20, 21]. In contrast, there is no evidence that autoreactive B cells are more sensitive than non-autoreactive B cells to CD20-based depletion. The clinical trials began in late 2001 and belimumab was finally approved in 2011, after the encouraging results of two randomized-controlled phase III trials (BLISS-52 and BLISS-76) [22, 23].

## Real-Life Experience with Belimumab in Refractory Active Systemic Lupus Erythematosus

Several authors investigated effectiveness and safety of belimumab among patients with SLE in clinical practice settings [24–34].

### The OBSErve Studies

OBSErve (evaluation Of use of Belimumab in clinical practice SEttings) is a multinational cohort study program designed to describe the clinical outcomes following belimumab therapy in a real-life setting. The results of these observational studies have been reported from the USA, Spain, Canada, and Germany so far [24–27].

The data collection was either purely retrospective [25–27] or a combination of a retrospective and a prospective phase [24]. The most common reasons for initiating belimumab therapy were ineffectiveness of patient’s previous treatment regimen (69.2–88% of patients, depending on the country), worsening of the patient’s condition (50–61%), and need to decrease corticosteroid drugs (40–67.3%). The primary outcome in all the OBSErve studies was the assessment of any changes in SLE disease activity by selected physicians. Disease activity, either global or organ-specific, was subjectively judged by the physician as worsened, not improved, minimally (< 20%) improved, clearly but moderately (20–49%) improved, greatly (50–79%) improved, or nearly normalized (at least 80% improvement). Other secondary outcomes included healthcare resource utilization, changes in validated disease activity scores (such as SELENA-SLEDAI and BILAG), steroid use, and laboratory tests.

*OBSErve US* study [24] was conducted in the USA in a cohort of 501 patients with SLE over 24 months in clinical practice setting (Table 1). The study was performed in two stages—from 6 months prior to first belimumab infusion (baseline) to 12 months post-baseline—data were collected retrospectively (with the exception of 284 patients for whom month 12 data were collected prospectively). Subsequently (months 18–24), patients were followed up prospectively. All patients received belimumab for at least 6 months.

**Table 1:**
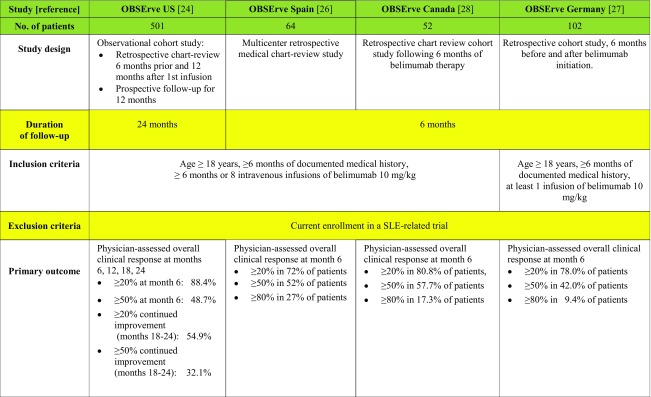

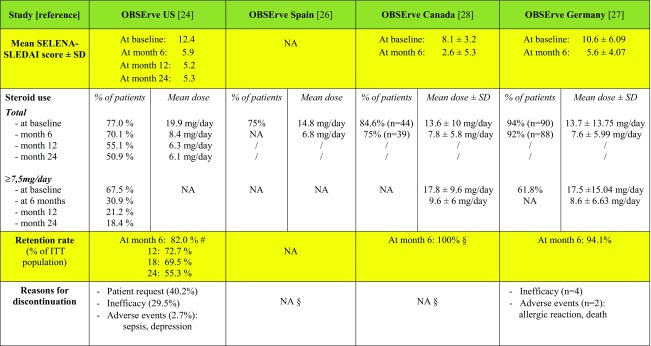
Summary of the OBSErve studies

According to physician assessment, at 6 months, 251 patients (88.4%) achieved a ≥ 20% improvement and 134 patients (48.7%) achieved a ≥ 50% improvement in overall clinical response to belimumab. No disease flares were reported at months 12, 18, and 24 in more than 99% of the responders. SELENA-SLEDAI [35] (Safety of Estrogen in Lupus Erythematosus National Assessment-Systemic Lupus Erythematosus Disease Activity Index) mean score decreased from 12.4 at baseline to 5.9 at month 6, and the lower score was maintained at months 12, 18, and 24, but since the number of patients assessed by SELENA-SLEDAI was small (*n* = 122), conclusions should be interpreted with caution. Notably, a decrease was seen in concomitant oral corticosteroid dose, i.e., from 19.9 to 6.8 mg/day at month 6. Laboratory tests showed that patients with normal levels of white blood cell count, platelet count, hemoglobin, C3, C4, ESR, and CRP at belimumab initiation remained normal throughout the study, while patients who had abnormal levels at baseline improved towards normal levels. The proportion of patients with anti-dsDNA antibody positivity decreased over the 24 months. By month 24, 112 (22.4%) patients were lost to follow-up and 112 (22.4%) discontinued belimumab treatment. The most common reasons for discontinuation were patient request (40.2%) and medication not effective (29.5%); 14 patients discontinued due to adverse events (AEs). A sensitivity analysis showed that patients who did not complete the 24-month follow-up less commonly displayed anti-dsDNA positivity and low complement at baseline compared with the 24-month completers.

*OBSErve Spain* [25] was a multicenter retrospective medical chart-review study that included 64 patients (Table 1). The overall clinical improvement at month 6 was ≥ 80, ≥ 50, and ≥ 20% in 27, 52, and 72% of patients, respectively; corticosteroid mean daily dose decreased from 14.8 to 6.8 mg (*p* < 0.001). Although the study remarked an increase in healthcare resource utilization for hematological (3.14 to 3.52; *p* = 0.045) and renal (5.95 to 6.59; *p* = 0.024) tests due to an increased frequency of follow-up visits, the treatment resulted cost-effective: There was a significant decrease in emergency-room visits (1.65 to 0.41; *p* = 0.001), unscheduled visits to treating-physician (1.02 to 0.03; *p* < 0.001), visits to other specialists (1.64 to 1.06; *p* = 0.017), and work absenteeism (25.6 to 5.7 days; *p* = 0.025) between the pre and post index periods.

*OBSErve Germany* [26] evaluated the effect of belimumab in 102 patients (Table 1). Data were collected from patient medical records 6 months before and after belimumab initiation. In addition to physician’s judgment of improvement, patients were evaluated with validated disease activity indexes (SELENA-SLEDAI, ECLAM-European Consensus Lupus Activity Measurement, Physician Global Assessment Scale, Patient Global Assessment Scale or BILAG assessment). After 6 months of belimumab treatment, 78% of patients showed an improvement in overall disease activity of at least 20% in their physician’s judgment; in 42% of patients, the improvement was at least 50%. Similar results were observed for arthritis, mucocutaneous manifestations, fatigue, low complement, and increased anti-dsDNA antibody levels. SELENA-SLEDAI decreased from 10.6 to 5.6 (*n* = 65), ECLAM score, Physician Global Assessment Scale, and Patient Global Assessment Scale also showing improvement. The mean daily prednisone equivalent dose of corticosteroids decreased from 13.7 to 7.6 mg overall, and from 17.5 to 8.6 mg in patients receiving a high corticosteroid dose at baseline. The discontinuation rate was low (six patients within 6 months), and in general, belimumab appeared to be well tolerated.

*OBSErve Canada* [27] was a retrospective multicenter medical chart review study of 52 patients with SLE who received at least eight infusions or 6 months of treatment with belimumab (Table 1). At month 6 after the first infusion of belimumab, the physician-determined clinical improvement was ≥ 20% in 80.8% of patients, ≥ 50% in 57.7% of patients, and ≥ 80% in 17.3% of patients. Response was judged to be inadequate in 19.2% of patients: Nine patients showed < 20% clinical improvement and one patient had no improvement; no patients were reported to have had a worsened disease. Corticosteroid mean dose decreased from 13.6 ± 10 to 7.8 ± 5.8 mg/day. AEs were reported in 12 patients; those that occurred in more than one patient were sinusitis (*n* = 3), diarrhea (*n* = 2), and headache (*n* = 2). Data regarding AEs that led to discontinuation within the first 6 months are not available.

In conclusion, all the OBSErve studies confirmed the phase III trial results regarding effectiveness and tolerability of belimumab. They also remarked the decrease in healthcare resource utilization, i.e., emergency-room visits, SLE-related hospitalizations, and unscheduled rheumatologic visits, after belimumab initiation. Only the number of scheduled physician visits increased, due to the monthly frequency of infusions.

As the authors stated, the study design has some limitations. In the absence of a control group, the reliability of the studies’ conclusions on belimumab is greatly weakened, as pre-treatment and post-treatment measurements cannot account for the natural course of this highly variable disease and part of the clinical response could be due to placebo effect. Furthermore, the quantification of clinical response was a non-standardized, subjective tool that has not been validated. Another limitation is in the inclusion criteria as USA, Spain, and Canada OBSErve studies required at least 6 months of treatment (≥ 8 doses) [24, 25, 27]. This strongly biases the study population in favor of those who well tolerated and responded to belimumab treatment, or were treated by physicians who were compliant with regular patient follow-up. OBSErve Germany, on the contrary, minimized selection bias by including all eligible patients at each site, and also if they discontinued belimumab therapy within the initial 6 months [26]. However, the study did not collect safety data besides those leading to withdrawal.

### Other Observational Clinical-Practice-Based Studies

Other groups investigated belimumab effectiveness and safety in the clinical practice setting [28–34].

*Hui-Yuen et al.* [28] evaluated belimumab effectiveness in SLE patients in a multicenter, observational, prospective cohort study (Table 2). Data were collected from 10 academic medical centers in the USA and Sweden. All 195 patients were required to have a diagnosis of SLE and have started treatment with belimumab. Patients with previous severe renal or neuropsychiatric involvement were excluded. Clinical response was defined using a simplified version of the SLE Responder Index [37], similar to the SLEDAI SRI-50 (see Table 2). Serological response was defined as a ≥ 25% improvement in the levels of C3, C4, and/or a 25% decrease in anti-dsDNA antibody levels. Initial clinical and serological response to belimumab was detected as early as 3 months after initiation of therapy as 52% of patients showed improvement in clinical manifestations that drove initiation of belimumab, 66% had at least a 25% increase in C3 values, and 48% had a 25% decrease in anti-dsDNA levels. Data were confirmed at 6 months, with 51% of patients showing a persistent clinical response and improvement in anti-dsDNA antibody and complement levels. The mean daily prednisone equivalent dose of corticosteroids decreased from 12.2 mg/day at baseline to 9.3 mg/day at 6 months, which may be clinically relevant though not statistically significant. Importantly, decrease in corticosteroid intake occurred especially in childhood-onset SLE, with 35% of patients being able to discontinue steroid treatment after the addition of belimumab to standard-of-care. In general, belimumab appeared to be well tolerated with 16% of patients who experienced adverse events.

**Table 2:**
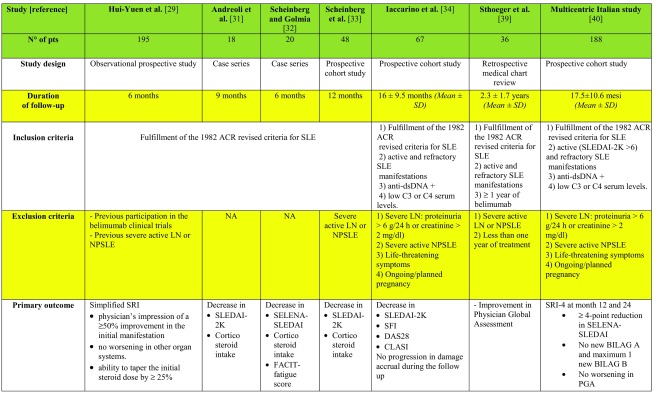

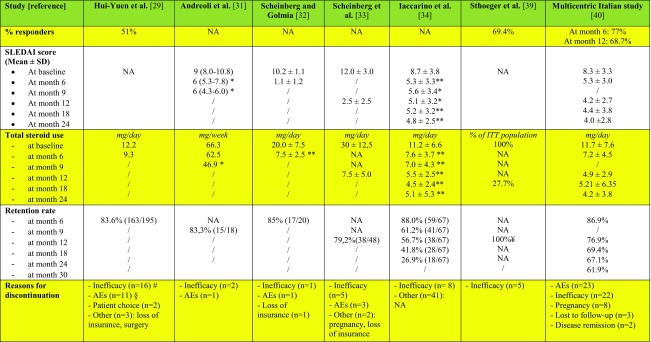
Summary of studies published so far about belimumab use in real-life after RCTs

*Andreoli et al.* [29] reported their experience with belimumab in the treatment of 18 patients with refractory SLE after the first year of licensed use in Italy (Table 2).

SLEDAI-2K showed a significant decrease from baseline to month 3 (*p* = 0.002), maintained also at month 6 and 9 (*p* = 0.012). The mean dose of prednisone administered required 9 months of therapy to show a significant decrease (*p* = 0.045), even though a trend towards decrease was noticed also at month 3 (77.2 vs. 80 mg/week) and 6 (65 vs. 80 mg/week). Changes in anti-dsDNA antibody and complement levels were not found to be significant; nevertheless, this lack of significance can be due to nearly half of the patients having low titers of anti-dsDNA and/or a slight reduction in complement levels at baseline. Patients with active serology, on the contrary, markedly improved after a few months of treatment. During the first 6 months, the administration of the drug was discontinued in three patients (16.7%): One patient discontinued the treatment due to recurrent upper respiratory tract infections, and two patients due to inefficacy. As the authors state, “treatment dropouts occurred in patients whose clinical history had always been characterized by closely repeated relapses” and “a patient who discontinued the drug due to ineffectiveness was not taking any concomitant therapy except for steroids due to intolerance to multiple drugs”. They also questioned whether a 6 months follow-up is adequate to assess whether belimumab is effective or not.

Two papers regarding the real-life experience with belimumab in Brazil have been published so far. The first one [30], published in 2014, analyzed the outcome of the treatment in patients with musculoskeletal symptoms in the first 6 months of treatment (Table 2). *Scheinberg and Golmia* evaluated 20 patients on standard of care and indication to receive belimumab (disease activity evaluated by SELENA-SLEDAI ≥ 8). A clear decrease in mean SELENA-SLEDAI score (from 10.2 ± 1.1 to 1.1 ± 1.2), mean corticosteroid dosage (from 20 to 7.5 mg/day), and anti-dsDNA antibody levels (from 180 to 60 IU/ml) was observed; C3 mean levels increased from 62 to 98 mg. A significant improvement on fatigue was also observed in the group of patients that completed 6 months of treatment, with the mean FACIT (Functional Assessment of Chronic Illness Therapy) score improving from 37.6 ± 3.8 to 48.8 ± 3.3, where a score of 52 represents zero fatigue.

A second article was published in 2016 by *Scheinberg et al.* [31] evaluating their real-life experience after 1 year on belimumab in 48 Brazilian patients (Table 2). Thirty-eight patients were still on treatment at the end of the year. A significant improvement was observed in the SLEDAI score, with a decrease from 12 ± 3.0 to 2.5 ± 2.5, as well as a partial improvement in serology and a trend for a decline in anti-DNA levels though not reaching statistical significance. Daily corticosteroid dose decreased from 30 ± 12.5 to 7.5 ± 5.0 mg.

During the first year, disease flares were observed in five patients requiring an increase in the corticosteroid dose. Two out of five patients received rituximab, another two were kept on a higher corticosteroid dose, and one was lost on follow-up. Adjustment of other concomitant immunosuppressants was not performed. After 1 year, 10 patients (16%) had treatment discontinued due to worsened disease activity (arthritis and rash exacerbation in three, anemia and thrombocytopenia in one, and severe constitutional symptoms in another one), unexpected pregnancy in one case, and side effects in three cases, i.e., severe rhinosinusitis after each infusion and uncontrolled itching after the second and third infusion, and one for loss of insurance coverage.

*Our group* [32] investigated effectiveness and safety of belimumab in a prospective cohort study. All 67 patients were required to fulfill the 1982 ACR revised criteria for SLE and have active/refractory disease manifestations, detectable anti-dsDNA antibodies, and low C3 or C4 serum levels; exclusion criteria were severe lupus nephritis (LN), severe active neuropsychiatric lupus (NPSLE), potentially life-threatening SLE manifestations, and ongoing or planned pregnancy. Clinical scores (i.e. SLEDAI-2K score, SELENA-SLEDAI Flare Index, SLICC-DI score), daily prednisone intake, and laboratory tests (i.e. white blood cells, anti-dsDNA antibody, C3 and C4 levels) were collected at baseline and at months 3, 6, 9, and 12 and then every 6 months. Skin manifestations were assessed by CLASIa (Cutaneous Lupus Erythematosus Disease Area and Severity Index activity) [38], arthritis by DAS28 (Disease Activity Score in 28 joints) [39], and renal involvement by serum creatinine levels, 24-h proteinuria, and urinary sediment. Mean SLEDAI-2K score [40] and anti-dsDNA antibody levels significantly decreased over time starting from month 3 (from 8.7 ± 3.8 to 6.1 ± 4.1 and from 102.4 ± 201 to 53.1 ± 69.0 IU/ml, respectively). At month 6, the total white blood cell count was significantly increased (from 2588 ± 910/mm^3^ at baseline to 3701 ± 1808/mm^3^), while prednisone daily intake and 24-h proteinuria were significantly decreased (from 11.2 ± 6.6 mg/day at baseline to 7.6 ± 3.7 mg/day and from 1.27 ± 0.68 at baseline to 0.88 ± 0.65, respectively). An increase in C3 and C4 serum levels, although not statistically significant, was also found. Regarding specific organ involvement, DAS28 score significantly (*p* < 0.001) decreased due to an improvement in patients with classic lupus polyarthritis (*p* < 0.001), but not in those with rheumatoid-like polyarthritis (i.e. persistent, deforming, erosive joint inflammation) (*p* = 0.08). Also, cutaneous manifestations had a good response to the drug, with a decrease in median CLASIa in both subacute cutaneous (SCLE) and acute cutaneous SLE (ACLE) manifestations. The flare rate 12 and 24 months before belimumab initiation was 78 flares/100 patients and 150 flares/100 patients, respectively. Nineteen flares were observed in 15 patients during follow-up, with a rate of 26 flares/100 patients/12 months and 44 flares/100 patients/24 months after belimumab initiation; the decrease in the flare rate was statistically significant in both periods (*p* = 0.001). Damage accrual, assessed with SDI (SLICC/ACR Damage Index) [41], remained unchanged during the 2-year follow-up after belimumab initiation. This may be due to the decrease in mean disease activity scores and in corticosteroid use in the population. Eight patients discontinued the treatment due to lack of efficacy. No severe adverse events were observed.

*Sthoeger et al.* [33] performed a retrospective medical chart review study of 36 Israeli patients treated with intravenous belimumab for at least 1 year. Disease response to belimumab was assessed by the Physicians Global Assessment. A 69.4% of patients had an excellent response, achieving clinical remission, 16.6% of patients improved with belimumab treatment without complete remission, and lack of response, i.e., disease flares or new disease manifestations during treatment, was observed in 13.9% of patients and led to drug discontinuation. Regarding serological abnormalities, normalization of anti-dsDNA antibodies and complement levels was observed in 48 and 50% of patients, respectively. The rate of patients treated with corticosteroids and immunosuppressants decreased from 100 to 27.7% and from 83.3 to 8.3%, respectively. There were neither severe adverse events nor discontinuations due to adverse events.

Lastly, a multicentric *prospective cohort study* [34] was conducted in Italy collecting data from 11 SLE referral centers. One hundred eighty-eight patients were enrolled in the study, with a mean follow-up period of 17.5 ± 10.6 months. The primary outcome measure was SRI-4 (SLE Responder Index-4) at months 12 and 24 (see Table 2).

Notably, 77.0 and 68.7% of patients achieved SRI-4 at month 12 and 24 of follow-up, respectively; 83.7% of responders at 12 months maintained the response at 24 months, while the majority of patients who did not achieve SRI-4 response at 12 months were non-responders also at 24 months. During belimumab treatment, C3 and C4 serum levels showed a significant increase, while SLEDAI-2K, prednisone daily use, 24 h proteinuria, DAS28, CLASI activity, and anti-dsDNA tested by ELISA significantly declined over time. By multivariate logistic regression analysis, high-dose (≥ 7.5 mg/day) corticosteroid therapy, SLEDAI-2K ≥ 10, and polyarthritis were baseline-independent predictors of SRI-4 response to belimumab. Conversely, immunosuppressive treatment at baseline was a negative predictor of response to belimumab (OR 0.11; 95%CI 0.01–0.89; *p* = 0.039), probably owing to a more severe, long-standing disease in patients who required immunosuppressants for disease control. Although both DAS28 and CLASIa significantly decreased during the follow-up, only polyarthritis was predictive of response to belimumab. Authors attribute this inconsistency to the structure of SELENA-SLEDAI score, which is part of the SLE Responder Index. Indeed, it is easier to obtain a ≥ 4-point reduction in SELENA-SLEDAI in patients with joint involvement than in those with mucocutaneous lesions, since polyarthritis alone scores 4 points, while inflammatory rash and mucosal ulcers count 2 points each.

Disease flares were assessed by SELENA-SLEDAI Flare Index (SFI). Researchers compared the mean number of flares/patient and the number of patients having at least one flare during the 12 and 24 months before and after belimumab initiation; they observed a significant decrease both in the number of flares/patient and in the number of patients having at least one flare after belimumab initiation, compared with the corresponding period before. Consistent with Bruce et al.’s study [36], conducted in 998 SLE patients who completed BLISS RCTs and entered in the long-term extension study, they observed a slowdown in organ damage accrual during follow-up. The main reasons for discontinuation were AEs (23 patients, 39.6%) and inadequate response (22 patients, 37.9%). Eight patients (13.8%) discontinued due to pregnancy/desire for pregnancy, two (3.4%) due to disease remission, and three (5.2%) were lost to follow-up. No correlation was found between baseline variables and belimumab withdrawal by univariate analysis. The main strengths of this study are the use of validated clinimetric and response measures, the long follow-up period, the large sample size, and the prospective collection of data. Conversely, limitations include the lack of a control population and the heterogeneous background treatment, since patients were treated in different centers.

### Safety and Tolerability

Complete safety data were reported only in some studies [27, 29, 32–34]. Adverse events occurred in a percentage ranging between 0.05 and 68.7% [27–30, 32–34], depending on the study design. Some studies [24, 26, 28, 31] reported only adverse events leading to belimumab withdrawal. To date, safety data regarding OBSErve Spain are still unavailable [25].

The most frequent adverse events were infections. In the OBSErve USA [24], four patients (0.07%) discontinued treatment due to sepsis, one patient due to pneumonia, one patient due to multiple infections, and one patient due to recurring infections. Andreoli et al. [29] observed infectious adverse events in seven cases (38.8% of patients) within 6 months, that is, four infections of the upper respiratory tract, in one case recurrent; two gastrointestinal infections, and one urinary tract infection. Recurrent upper respiratory tract infections led one patient to drug discontinuation; however, no definite attribution to drug administration could be made as the patient was concomitantly taking multiple immunosuppressive drugs and a high steroid dosage, often increased by the patient herself without medical consent.

Also, Scheinberg et al. [31] and OBSErve Canada [27] reported sinusitis as adverse event in two and three patients, respectively. Hui-Yuen et al. [28] reported seven cases of infection (3.6% of patients). The most severe was Group A Streptococcal bacteremia in a patient who had an elective cervical lymph node biopsy performed 5 days prior to development of bacteremia. Besides, pneumonia and axillary *methicillin*-resistant *Staphylococcus aureus* infection were reported.

In our group’s study [32], 34 patients (50.7%) developed non-severe infections (21 upper respiratory tract infections, 21 urinary tract infections, 10 gastroenteritis, 4 vaginal candidosis, 2 labial Herpes simplex (HSV), 2 conjunctivitis, 1 relapse of genital HSV, 2 dental infections, 1 skin infection, 1 orchiepididymitis, 1 cutaneous HSV) and one patient suffered from a severe pneumonitis. In Sthoeger et al.’s cohort [33], four patients (11.1%) developed infections (one pneumonia and three Herpes zoster), which were considered probably related to belimumab treatment.

In the multicentric Italian study [34], 101 patients (53.7%) developed non-severe infections (89 upper respiratory tract infections, 53 urinary tract infections, 38 gastroenteritis, 19 flu, 14 low respiratory tract infections, 14 relapses of vaginal HSV, 11 vaginal candidosis, 11 skin infections, 10 labial HSV, 9 conjunctivitis, 4 dental infections, 1 orchiepididymitis).

It should be noted that in the absence of a control group, it is difficult to distinguish whether the infections are directly attributable to belimumab or not. Most of the patients, indeed, are treated with corticosteroids and/or immunosuppressants, which increase vulnerability to infections.

Regarding hypersensitivity and infusion-site reactions, in Scheinberg’s cohort [31], one patient discontinued treatment due to uncontrolled itching after the second and third infusion. Other three patients had mild/moderate hypersensitivity and infusion-site reactions that did not lead to a discontinuation; two of the patients received pretreatment with antihistamine drugs prior to the next belimumab infusion, with improvement. Hui-Yuen et al. [28] reported three infusion reactions (1.5% of patients). In the OBSErve Germany [26], one patient (0.9% of patients) discontinued treatment due to an allergic reaction. Our group [32] reported a 3% of patients with infusion reactions (*n* = 2) and a 19.4% of patients with hypersensitivity reactions (*n* = 13). In the multicentric Italian study [34], 26 patients (13.8%) experienced at least one hypersensitivity reaction and 4 patients (2.1%) at least one infusion reaction. Four patients withdrew from treatment due to infusion reactions and one due to hypersensitivity reactions.

Some of the studies remarked the development or worsening of neuropsychiatric SLE while taking belimumab. Hui-Yuen et al. [28] reported one patient with stroke, one with new-onset psychosis, one with severe depression, one with new-onset seizures, and two with worsening of neuropsychiatric SLE. In all these patients, belimumab was discontinued. Depression was the cause of withdrawal for three patients in the OBSErve USA study [24] and one patient in the multicentric Italian study [34].

Sporadic severe adverse events that took place after the beginning of belimumab therapy include myocardial infarction [28], lupus mielopathy [26], lower limb deep vein thrombosis [32, 34], severe leukopenia [34], and pulmonary embolism [34]. In the OBSErve Germany [26], one patient died during the study due to undiagnosed cardiomyopathy; in the OBSErve USA [24], one patient died due to central nervous system lupus (both not suspected of being belimumab related). In the multicentric Italian study [34], one patient was diagnosed with thyroid cancer. No deaths nor malignancies were reported in all other studies. As for infections, it is difficult to establish whether an adverse event is directly attributable to belimumab in a short-period observation: SLE patients often have comorbidities, e.g., antiphospholipid syndrome, and take multiple medications. However, to date, no specific pattern of adverse events was observed.

## Belimumab in the Current Management of SLE: Expert Opinion

According to the EULAR recommendations for the management of SLE [36], standard treatment includes hydroxycloroquine, when not strongly contraindicated, and low-dose prednisone. Immunosuppressants, e.g., azathioprine, mycophenolate mofetil, and methotrexate, should be considered in refractory cases or when steroid dose cannot be reduced in the long-term use.

When EULAR recommendations were published, belimumab was not available. Nowadays, according to RCTs and real-life experience, belimumab can be proposed to patients with arthritis and skin rash/mucocutaneous manifestations, active disease, especially with a relapsing-remitting pattern, active serology, and high prednisone intake, particularly if they are young females reluctant to use immunosuppressants. The earlier the use of belimumab in SLE course, the better the clinical response, as logically expected. Alternatively, belimumab may be used as an add-on to treatment when refractory manifestations persist despite the use of immunosuppressants [42].

## Potential Off-Label Uses of Belimumab

To date, belimumab is indicated only in the treatment of adults with active and autoantibody-positive SLE who are receiving standard therapy. However, soluble BLyS was found elevated in the sera of patients with several immune-based diseases, including primary Sjögren’s syndrome [43], ANCA-associated vasculitis [44], rheumatoid arthritis [17], idiopathic inflammatory myopaties [45], IgA nephropathy [46], and multiple sclerosis [47]. Thus, the downregulation of BLyS by belimumab might be potentially useful in all these disorders.

Good results were reported from the open-label phase II studies of 30 patients with primary Sjögren’s syndrome [48, 49] and the double-blind placebo-controlled pilot study of 20 patients with early diffuse cutaneous systemic sclerosis [50].

Actually, additional therapeutic indications are under investigation including idiopathic membranous glomerulonephritis (ClinicalTrials.gov: NCT01610492), myasthenia gravis (NCT01480596), symptomatic Waldenstrom’s macroglobulinemia (ClinicalTrials.gov: NCT01142011), and chronic immune thrombocytopenia (ClinicalTrials.gov: NCT01440361).

## Conclusions

Consistent with the results of the phase III clinical trials [22, 23], all studies remarked that belimumab provides additional benefits when used in patients with refractory SLE, allowing a higher rate of patients to reach clinical or complete remission and to taper or discontinue corticosteroids.

Prolonged remission is associated with a decrease in damage progression [51–54] and, therefore, better outcomes. Regarding the predictors of treatment efficacy, real-life experience [42] confirmed the results of the pooled post hoc analysis of the phase III clinical trials [55]: Patients who are more likely to respond to belimumab display an active disease with acute mucocutaneous and musculoskeletal manifestations, active serology, and high prednisone intake (≥ 7.5 mg/day) with a relapsing-remitting course.

The main limitations of the studies we considered in our review include the selection criteria, the lack of a control group, and the lack of precise restrictions regarding concomitant medication management.

In the absence of a control group, it is difficult to draw definite conclusions on the real belimumab effectiveness since part of the clinical response could be due to the placebo effect. In addition, the lack of restrictions regarding background therapy may be a confounding factor, as clinical improvements could be due to changes in concomitant medications and not to belimumab. Some of the studies excluded patients who discontinued belimumab within 6 [24, 25, 27] or 12 months [33]; this strongly biased the study population in favor of those who tolerated and responded to the treatment, limiting the possibility to make comparisons with other studies.

Another limitation is the short period of observation, i.e., 6–24 months depending on the study design. SLE is a highly variable disease; a 6-month follow-up [as seen in 26–29, 32] may be inadequate for patients with a clinical history of disease flares followed by long periods of remission.

This notwithstanding, these clinical-practice-based studies provided us a realistic picture of real-life use of belimumab: The high retention rates at 6, 9, and 12 months (82–94.1, 61.2–83.3, and 56.7–79.2%, respectively) reflect its clinical effectiveness and tolerability, in the absence of significant adverse events.
