# Correction to: Effectiveness, Tolerability, and Safety of Belimumab in Patients with Refractory SLE: a Review of Observational Clinical-Practice-Based Studies

**DOI:** 10.1007/s12016-018-8704-1

**Published:** 2018-08-23

**Authors:** Francesca Trentin, Mariele Gatto, Margherita Zen, Maddalena Larosa, Linda Nalotto, Francesca Saccon, Elisabetta Zanatta, Luca Iaccarino, Andrea Doria

**Affiliations:** Division of Rheumatology, University of Padova, Via Giustiniani, 2, 35128 Padova, Italy

**Correction to: Clinic Rev Allerg Immunol (2018) 54:331–343**

10.1007/s12016-018-8675-2

The original version of this article unfortunately contained mistakes. The changes are as follows:1. Maddalena Larosa’s given name and surname were inadvertently interchanged. The correct presentation is shown above.2. The article [Effectiveness, Tolerability, and Safety of Belimumab in Patients with Refractory SLE: a Review of Observational Clinical-Practice-Based Studies], written by [Francesca Trentin, Mariele Gatto, Margherita Zen, Maddalena Larosa, Linda Nalotto, Francesca Saccon, Elisabetta Zanatta, Luca Iaccarino, and Andrea Doria], was originally published electronically on the publisher’s internet portal (currently SpringerLink) on 06 March 2018 without open access.

With the author (s)’ decision to opt for Open Choice the copyright of the article changed on August 2018 to © The Author (s) 2018 and the article is forthwith distributed under the terms of the Creative Commons Attribution 4.0 International License (http://creativecommons.org/licenses/by/4.0/), which permits use, duplication, adaptation, distribution and reproduction in any medium or format, as long as you give appropriate credit to the original author (s) and the source, provide a link to the Creative Commons license and indicate if changes were made.

